# Supplementary material for: Additional Value of Patient-Reported Symptom Monitoring in Cancer Care: A Systematic Review of the Literature
Source: Cancers (Basel). 2021 Sep 15;13(18):4615. doi: 10.3390/cancers13184615 (PMC8469093; doi:10.3390/cancers13184615)
Supplement: Supplementary file 1 [file cancers-13-04615-s001.zip › cancers-1344185-supplementary.pdf]

cancers

MDPI

Supplementary Material

Additional Value of Patient-Reported Symptom Monitoring in Cancer Care: A Systematic Review of the Literature

Luís Lizán, Lucía Pérez-Carbonell and Marta Comellas

Table S1. PRISMA checklist [1].

Section and Topic	Item #	Checklist Item	Location where Item Is Reported
**TITLE**			
Title	1	Identify the report as a systematic review.	Page 1; tile; "Additional value of patient-reported symptom monitoring in cancer care: a systematic review of the literature"
**ABSTRACT**			
Abstract	2	See the PRISMA 2020 for Abstracts checklist.	See the Supplementary table 5 (PRISMA checklist for abstracts)
**INTRODUCTION**			
Rationale	3	Describe the rationale for the review in the context of existing knowledge.	Pages 2 and 3; Introduction section, from "Cancer patients suffer from significant physical and psychosocial consequences" to "there is insufficient understanding of the impact of patient-reported symptom monitoring on health outcomes"
Objectives	4	Provide an explicit statement of the objective(s) or question(s) the review addresses.	Page 2; Introduction, "Therefore, this systematic review aimed to describe the benefits of patient-reported symptom monitoring —regardless of the modality (paper-based or electronic) on health outcomes such as clinical (e.g., survival), patient-reported (e.g., Health-Related Quality of Life [HRQoL], general perception or feelings of well-being, satisfaction, etc.) and economic outcomes (use of healthcare resources, costs, cost-effectiveness, etc.)"
**METHODS**			
Eligibility criteria	5	Specify the inclusion and exclusion criteria for the review and how studies were grouped for the syntheses.	Page 3; Methods section 2.1., second paragraph, "The search was limited to original articles…." Page 3: Table 1 (Eligibility criteria defined by PICOS)
Information sources	6	Specify all databases, registers, websites, organisations, reference lists and other sources searched or consulted to identify studies. Specify the date when each source was last searched or consulted.	Pages 2 and 3; Methods section 2.1., "the search was adapted to the Medline/PubMed international database" to "And reviewed international congress pages related to outcomes search and pharmacoeconomics, such as the

Section and Topic	Item #	Checklist Item	Location where Item Is Reported
			International Society for Pharmacoeconomics and Out-comes Research (ISPOR).”
Search strategy	7	Present the full search strategies for all databases, registers and websites, including any filters and limits used.	Page 2 and 3; Methods section 2.1., “We conducted a systematic review of the literature ….” Supplementary Tables 1 (Comprehensive list of search terms considered) and 2 (search strategy)
Selection process	8	Specify the methods used to decide whether a study met the inclusion criteria of the review, including how many reviewers screened each record and each report retrieved, whether they worked independently, and if applicable, details of automation tools used in the process.	Page 3; Methods section 2.2., first paragraph, “Two researchers independently screened each of the identified publications based on titles, abstracts, and full texts for inclusion criteria. Any discrepancies between re-viewers were resolved through consensus and, if necessary, by consulting a third re-viewer”
Data collection process	9	Specify the methods used to collect data from reports, including how many reviewers collected data from each report, whether they worked independently, any processes for obtaining or confirming data from study investigators, and if applicable, details of automation tools used in the process.	-
Data items	10a	List and define all outcomes for which data were sought. Specify whether all results that were compatible with each outcome domain in each study were sought (e.g. for all measures, time points, analyses), and if not, the methods used to decide which results to collect.	Page 3; Methods section 2.2., second paragraph : “From the final selected articles, we extracted the following variables….“
	10b	List and define all other variables for which data were sought (e.g. participant and intervention characteristics, funding sources). Describe any assumptions made about any missing or unclear information.	-
Study risk of bias assessment	11	Specify the methods used to assess risk of bias in the included studies, including details of the tool(s) used, how many reviewers assessed each study and whether they worked independently, and if applicable, details of automation tools used in the process.	-
Effect measures	12	Specify for each outcome the effect measure(s) (e.g. risk ratio, mean difference) used in the synthesis or presentation of results.	-
Synthesis methods	13a	Describe the processes used to decide which studies were eligible for each synthesis (e.g. tabulating the study intervention characteristics and comparing against the planned groups for each synthesis (item #5).	-
	13b	Describe any methods required to prepare the data for presentation or synthesis, such as handling of missing summary statistics, or data conversions.	-

Section and Topic	Item #	Checklist Item	Location where Item Is Reported
	13c	Describe any methods used to tabulate or visually display results of individual studies and syntheses.	-
	13d	Describe any methods used to synthesize results and provide a rationale for the choice(s). If meta-analysis was performed, describe the model(s), method(s) to identify the presence and extent of statistical heterogeneity, and software package(s) used.	-
	13e	Describe any methods used to explore possible causes of heterogeneity among study results (e.g. subgroup analysis, meta-regression).	-
	13f	Describe any sensitivity analyses conducted to assess robustness of the synthesized results.	-
Reporting bias assessment	14	Describe any methods used to assess risk of bias due to missing results in a synthesis (arising from reporting biases).	-
Certainty assessment	15	Describe any methods used to assess certainty (or confidence) in the body of evidence for an outcome.	Page 3; Methods section 2.2., third paragraph : "Two researchers independently assessed the quality of the studies…."
		**RESULTS**	
Study selection	16a	Describe the results of the search and selection process, from the number of records identified in the search to the number of studies included in the review, ideally using a flow diagram.	Page 4; Results section 3.1., first paragraph: "We first identified a total of 1248 studies in MedLine/PubMed. Of these, 1190 …." Page 4; Figure 1 (PRISMA flow diagram)
	16b	Cite studies that might appear to meet the inclusion criteria, but which were excluded, and explain why they were excluded.	Page 4; Results section 3.1., first paragraph: From: "Supplementary Table 3 specifies the articles excluded and the reasons for their exclusion" Supplementary Table 3 (Excluded studies)
Study characteristics	17	Cite each included study and present its characteristics.	Page 16; Results section. From "In short, we identified 16 publications from 13 different studies: three of them published results from the same clinical trial reported" to "most of which evaluated the impact of patient-reported symptom monitoring on lung cancer patients". Pages 6 to 15, Table 2 (Summary of the identified studies)
Risk of bias in studies	18	Present assessments of risk of bias for each included study.	Pages 6 to 15, Table 2 (Summary of the identified studies) column study quality
Results of individual studies	19	For all outcomes, present, for each study: (a) summary statistics for each group (where appropriate) and (b) an effect estimate and its precision (e.g. confidence/credible interval), ideally using structured tables or plots.	Pages 6 to 15, Table 2 (Summary of the identified studies)
Results of syntheses	20a	For each synthesis, briefly summarise the characteristics and risk of bias among contributing studies.	-
	20b	Present results of all statistical syntheses conducted. If meta-analysis was done, present for each the summary estimate and its precision (e.g.	-

Section and Topic	Item #	Checklist Item	Location where Item Is Reported
		confidence/credible interval) and measures of statistical heterogeneity. If comparing groups, describe the direction of the effect.	
	20c	Present results of all investigations of possible causes of heterogeneity among study results.	-
	20d	Present results of all sensitivity analyses conducted to assess the robustness of the synthesized results.	-
Reporting biases	21	Present assessments of risk of bias due to missing results (arising from reporting biases) for each synthesis assessed.	-
Certainty of evidence	22	Present assessments of certainty (or confidence) in the body of evidence for each outcome assessed.	-
**DISCUSSION**			
Discussion	23a	Provide a general interpretation of the results in the context of other evidence.	Page 19-21; discussion section
	23b	Discuss any limitations of the evidence included in the review.	Page 19-21; discussion section
	23c	Discuss any limitations of the review processes used.	Page 21; discussion section; Study limitations subsection (4.2)
	23d	Discuss implications of the results for practice, policy, and future research.	Page 21; discussion section; Other implications of patient-reported symptom monitoring (4.1)
**OTHER INFORMATION**			
Registration and protocol	24a	Provide registration information for the review, including register name and registration number, or state that the review was not registered.	-
	24b	Indicate where the review protocol can be accessed, or state that a protocol was not prepared.	-
	24c	Describe and explain any amendments to information provided at registration or in the protocol.	-
Support	25	Describe sources of financial or non-financial support for the review, and the role of the funders or sponsors in the review.	Page 22; "Funding: This research received no external funding"
Competing interests	26	Declare any competing interests of review authors.	-
Availability of data, code and other materials	27	Report which of the following are publicly available and where they can be found: template data collection forms; data extracted from included studies; data used for all analyses; analytic code; any other materials used in the review.	-

Table S2. PRISMA checklist for abstracts.

Section and topic	Item #	Checklist item	Location where item is reported
**Title**			
Title	1	Identify the report as a systematic review.	Page 1; "We conducted a systematic literature review"
**Background**			
Objectives	2	Provide an explicit statement of the main objective(s) or question(s) the review addresses.	Page 1; "To describe the benefit of patient-reported symptom monitoring on clinical, other patient-reported, and economic outcomes"
Methods			
Eligibility criteria	3	Specify the inclusion and exclusion criteria for the review.	Page 1; "The search was limited to original articles published between 2011 and 2021 in English and Spanish."
Information sources	4	Specify the information sources (e.g., databases, registers) used to identify studies and the date when each was last searched.	Page 1; "We conducted a systematic literature review using Medline/PubMed"
Risk of bias	5	Specify the methods used to assess risk of bias in the included studies.	-
Synthesis of results	6	Specify the methods used to present and synthesise results.	-
**Results**			
Included studies	7	Give the total number of included studies and participants and summarise relevant characteristics of studies.	Page 1; "We identified 16 reports that deal with the benefit of patient-reported symptom monitoring (col-lected mostly electronically) on different outcomes."
Synthesis of results	8	Present results for main outcomes, preferably indicating the number of included studies and participants for each. If meta-analysis was done, report the summary estimate and confidence/credible interval. If comparing groups, indicate the direction of the effect (i.e. which group is favoured).	Page 1; Results' paragraph. From "We identified 16 reports that deal with the benefit of patient-reported symptom monitoring (col-lected mostly electronically) on different outcomes" to "Additionally, six studies observed that this monitoring approach prevented unplanned emergency room visits and hospital readmissions, leading to a substantial decrease in healthcare usage"
**Discussion**			
Limitations of evidence	9	Provide a brief summary of the limitations of the evidence included in the review (e.g. study risk of bias, inconsistency and imprecision).	-
Interpretation	10	Provide a general interpretation of the results and important implications.	Page 1; Conclusion's paragraph.
**Other**			
Funding	11	Specify the primary source of funding for the review.	-
Registration	12	Provide the register name and registration number.	-

Table S3. Comprehensive list of search terms considered.

Comprehensive list of search terms considered			
**Disease**	**PROs (measurement)**	**Assessment**	**Benefits**
Neoplasm*<br>Cancer<br>neoplasia*<br>Malignanc*<br>Oncolog*	"Patient Reported Outcome Measures"<br>"Patient Reported Outcome*"<br>"patient reported symptom*"<br>"patient reported performance"<br>"Patient Outcome Assessment"<br>"Patient-Centered Care"<br>"Health status"<br>Symptom*<br>"adverse event*"<br>well-being<br>functional<br>functioning<br>performance	Assessment<br>Evaluation<br>Monitoring<br>Self-reported<br>self-report<br>self-rated	Progression<br>Survival<br>"Treatment Outcome"<br>"Response Evaluation Criteria in Solid Tumors"<br>"patient satisfaction"[Mesh]<br>"Treatment Adherence and Compliance"[Mesh]<br>"patient safety"[Mesh]<br>"adverse event*"<br>"clinical utility"<br>"clinical benefit"<br>"clinical impact"<br>"clinical relevance"<br>"clinical significance"<br>"clinical performance"<br>"clinical implication*"<br>"clinical meaningfulness"<br>Economics [Mesh]<br>Economic*<br>Cost*<br>price<br>pharmacoeconomic*<br>"Health Resources" [Mesh]

Table S4. Search strategy.

Search strategies used by database				
**Database**	**Search Dates**	**Languages**	**Articles type**	Search Strategy
MEDLINE	Last 10 years	Abstract available in English/Spanish	Any	(Neoplasm*[ti] OR cancer[ti] OR neoplasia*[ti] OR Malignanc*[ti] OR oncolog*[ti]) AND ("Patient Reported Outcome Measures"[ti] OR "Patient Reported Outcome*"[ti] OR “patient reported symptom*”[ti] OR “patient reported performance”[ti] OR "Patient Outcome Assessment"[ti] OR "Patient-Centered Care"[ti] OR (("Health status"[ti] OR Symptom*[ti] OR “adverse event*”[ti] OR well-being[ti] OR functional[ti] OR functioning[ti] OR performance[ti]) AND (Assessment[ti] OR Evaluation[ti] OR Monitoring[ti] OR Self-reported[ti] OR self-report[ti] OR self-rated[ti]))) AND (Progression OR survival OR “Treatment Outcome” OR “Response Evaluation Criteria in Solid Tumors” OR “patient satisfaction”[Mesh] OR “Treatment Adherence and Compliance”[Mesh] OR “patient safety”[Mesh] OR “adverse event*” OR ((clinical) AND (utility OR benefit OR impact OR relevance OR significance OR performance OR implication* OR meaningfulness))) AND (Economic* OR Economics [Mesh] OR cost* OR price OR pharmacoeconomic* OR "Health Resources" [Mesh])

Table S5. Excluded studies.

Excluded Studies	Reason for Exclusion
An J, et al. [2]	It does not assess the impact of patient-reported symptoms monitoring on relevant outcomes
Andikyan V, et al. [3]	It is focused on feasibility and utility of PROs
Baeksted CW, et al. [4]	It does not assess the impact of patient-reported symptoms monitoring on relevant outcomes
Basch E, et al. [5]	It does not assess the impact of patient-reported symptoms monitoring on relevant outcomes
Basch E, et al. [6]	It is not an original study
Basch E, et al. [7]	It is not an original study
Basch E, et al. [8]	It does not assess the impact of patient-reported symptoms monitoring on relevant outcomes
Boysen ME, et al. [9]	It does not assess the impact of patient-reported symptoms monitoring on relevant outcomes
Danner et al. [10]	It does not assess the impact of patient-reported symptoms monitoring on relevant outcomes
de Rooij BH, et al. [11]	It does not assess patient-reported symptoms surveillance
de Souza JA, et al. [12]	It is focused on the development of a PROM
Denis F, et al. [13]	It does not assess the impact of patient-reported symptoms monitoring on relevant outcomes
Friis RB, et al. [14]	It is focused on feasibility and utility of PROs
Gressel GM, et al. [15]	It does not assess the impact of patient-reported symptoms monitoring on relevant outcomes
Hauth et al. [16]	It is focused on feasibility and utility of PROs
Heyn L, et al. [17]	It is not an original study
Hoque DME, et al. [18]	It does not assess the impact of patient-reported symptoms monitoring on relevant outcomes
Hsu T, et al. [19]	It is focused on feasibility and utility of PROs
Kargo AS, et al. [20]	It is not available for full-text assessment
Lagendijk M, et al. [21]	It is not an original study
Marino D, et al. [22]	It does not assess the impact of patient-reported symptoms monitoring on relevant outcomes
McGee SF, et al. [23]	It does not assess the impact of patient-reported symptoms monitoring on relevant outcomes
Mooney KH, et al. [24]	It does not assess the impact of patient-reported symptoms monitoring on relevant outcomes
Montgomery N, et al. [25]	It is focused on feasibility and utility of PROs
Nicolaije KA, et al. [26]	It does not assess the impact of patient-reported symptoms monitoring on relevant outcomes
Palmer SC, et al. [27]	It does not assess the impact of patient-reported symptoms monitoring on relevant outcomes
Richards HS, et al.[28]	It is focused on feasibility and utility of PROs
Rossi LA, et al. [29]	It is focus on PROM as a predictive factor
Smith TG, et al. [30]	It does not assess the impact of patient-reported symptoms monitoring on relevant outcomes
Smith SK, et al. [31]	It does not assess the impact of patient-reported symptoms monitoring on relevant outcomes
Stormoen DR, et al. [32]	It does not assess the impact of patient-reported symptoms monitoring on relevant outcomes
Stukenborg GJ, et al. [33]	It is focus on PROM as a predictive factor
Suh SY, e t al. [34]	It is focus on PROM as a predictive factor
Sutradhar R, et al. [35]	It is focus on PROM as a predictive factor

Taarnhøj GA, et al. [36]	It is focused on feasibility and utility of PROs
Takeuchi EE, et al. [37]	It is focused on patient-physician communication
van Egdom LES, et al. [38]	It does not assess the impact of patient-reported symptoms monitoring on relevant outcomes
Vistad I. et al. [39]	It is not an original study
Wanat M, et al. [40]	It is focused on feasibility and utility of PROs
Whitehead L, et al. [41]	It does not assess the impact of patient-reported symptoms monitoring on relevant outcomes
Wright AA, et al. [42]	It is focused on feasibility and utility of PROs
Yogananda MN, et al. [43]	It does not assess the impact of patient-reported symptoms monitoring on relevant outcomes
Zebralla V, et al. [44]	It does not assess the impact of patient-reported symptoms monitoring on relevant outcomes
Zylla DM, et al. [45]	It is focused on feasibility and utility of PROs

References

1. Page, M.J.; E McKenzie, J.; Bossuyt, P.M.; Boutron, I.; Hoffmann, T.C.; Mulrow, C.D.; Shamseer, L.; Tetzlaff, J.M.; A Akl, E.; E Brennan, S.; et al. The PRISMA 2020 statement: an updated guideline for reporting systematic reviews. *BMJ* **2021**, *372*, n71, https://doi.org/10.1136/bmj.n71.
2. An, J.; Lau, A. Economic evaluation of patient-centered care among long-term cancer survivors.. *Am. J. Manag. care* **2018**, *24*, e292–e299.
3. Andikyan, V.; Rezk, Y.; Einstein, M.H.; Gualtiere, G.; Leitao, M.M.; Sonoda, Y.; Abu-Rustum, N.R.; Barakat, R.R.; Basch, E.M.; Chi, D.S. A prospective study of the feasibility and acceptability of a Web-based, electronic patient-reported outcome system in assessing patient recovery after major gynecologic cancer surgery. *Gynecol. Oncol.* **2012**, *127*, 273–277, https://doi.org/10.1016/j.ygyno.2012.07.124.
4. Baeksted, C.W.; Nissen, A.; Knoop, A.; Christiansen, M.; Pappot, H. Handling of symptomatic adverse events in breast cancer patients receiving adjuvant chemotherapy in a cluster randomized trial with electronic patient-reported outcomes as intervention. *Breast J.* **2019**, *25*, 1295–1296, https://doi.org/10.1111/tbj.13477.
5. Basch, E.; Stover, A.M.; Schrag, D.; Chung, A.; Jansen, J.; Henson, S.; Carr, P.; Ginos, B.; Deal, A.; Spears, P.A.; et al. Clinical Utility and User Perceptions of a Digital System for Electronic Patient-Reported Symptom Monitoring During Routine Cancer Care: Findings From the PRO-TECT Trial. *JCO Clin. Cancer Informatics* **2020**, *4*, 947–957, https://doi.org/10.1200/cci.20.00081.
6. Basch, E.; Charlot, M.; Dueck, A.C. Population-level evidence of survival benefits of patient-reported outcome symptom monitoring software systems in routine cancer care. *Cancer Med.* **2020**, *9*, 7797–7799, https://doi.org/10.1002/cam4.3480.
7. Basch, E.; Leahy, A.B.; Dueck, A.C. Benefits of Digital Symptom Monitoring With Patient-Reported Outcomes During Adjuvant Cancer Treatment. *J. Clin. Oncol.* **2021**, *39*, 701–703, https://doi.org/10.1200/jco.20.03375.
8. Basch, E.; A Wood, W.; Schrag, D.; Sima, C.S.; Shaw, M.; Rogak, L.J.; Kris, M.G.; Shouery, M.; Bennett, A.; Atkinson, T.; et al. Feasibility and clinical impact of sharing patient-reported symptom toxicities and performance status with clinical investigators during a phase 2 cancer treatment trial. *Clin. Trials* **2015**, *13*, 331–337, https://doi.org/10.1177/1740774515615540.
9. Boysen, M.E.; Zätterström, U.K.; Evensen, J.F. Self-reported Symptoms to Monitor Recurrent Head and Neck Cancer-Analysis of 1,678 Cases.. *Anticancer. Res.* **2016**, *36*.
10. Danner, M.; Hung, M.-Y.; Yung, T.M.; Ayoob, M.; Lei, S.; Collins, B.T.; Suy, S.; Collins, S.P. Utilization of Patient-Reported Outcomes to Guide Symptom Management during Stereotactic Body Radiation Therapy for Clinically Localized Prostate Cancer. *Front. Oncol.* **2017**, *7*, 227, https://doi.org/10.3389/fonc.2017.00227.
11. De Rooij, B.H.; Ezendam, N.P.M.; Mols, F.; Vissers, P.A.J.; Thong, M.S.Y.; Vlooswijk, C.C.P.; Oerlemans, S.; Husson, O.; Horevoorts, N.J.E.; Van De Poll-Franse, L.V. Cancer survivors not participating in observational patient-reported outcome studies have a lower survival compared to participants: the population-based PROFILES registry. *Qual. Life Res.* **2018**, *27*, 3313–3324, https://doi.org/10.1007/s11136-018-1979-0.
12. De Souza, J.A.; Yap, B.J.; Hlubocky, F.J.; Wroblewski, K.; Ratain, M.J.; Cella, D.; Daugherty, C.K. The development of a financial toxicity patient-reported outcome in cancer: The COST measure. *Cancer* **2014**, *120*, 3245–3253, https://doi.org/10.1002/cncr.28814.

13. Denis, F.; Viger, L.; Charron, A.; Voog, E.; Dupuis, O.; Pointreau, Y.; Letellier, C. Detection of lung cancer relapse using self-reported symptoms transmitted via an Internet Web-application: pilot study of the sentinel follow-up. *Support. Care Cancer* **2014**, *22*, 1467–1473, https://doi.org/10.1007/s00520-013-2111-1.
14. Friis, R.B.; I Hjollund, N.H.; Mejdahl, C.T.; Pappot, H.; Skuladottir, H. Electronic symptom monitoring in patients with metastatic lung cancer: a feasibility study. *BMJ Open* **2020**, *10*, e035673, https://doi.org/10.1136/bmjopen-2019-035673.
15. Gressel, G.M.; Dioun, S.M.; Richley, M.; Lounsbury, D.W.; Rapkin, B.D.; Isani, S.; Nevadunsky, N.S.; Kuo, D.; Novetsky, A.P. Utilizing the Patient Reported Outcomes Measurement Information System (PROMIS®) to increase referral to ancillary support services for severely symptomatic patients with gynecologic cancer. *Gynecol. Oncol.* **2019**, *152*, 509–513, https://doi.org/10.1016/j.ygyno.2018.10.042.
16. Hauth, F.; Bizu, V.; App, R.; Lautenbacher, H.; Tenev, A.; Bitzer, M.; Malek, N.P.; Zips, D.; Gani, C. Electronic Patient-Reported Outcome Measures in Radiation Oncology: Initial Experience After Workflow Implementation. *JMIR mHealth uHealth* **2019**, *7*, e12345, https://doi.org/10.2196/12345.
17. Heyn, L.; Finset, A.; Ruland, C.M. Talking About Feelings and Worries in Cancer Consultations. *Cancer Nurs.* **2013**, *36*, E20–E30, https://doi.org/10.1097/ncc.0b013e318254af66.
18. Hoque, D.M.E.; Earnest, A.; Ruseckaite, R.; Lorgelly, P.; Sampurno, F.; Evans, M.; Evans, S.M. A randomised controlled trial comparing completeness of responses of three methods of collecting patient-reported outcome measures in men diagnosed with prostate cancer. *Qual. Life Res.* **2018**, *28*, 687–694, https://doi.org/10.1007/s11136-018-2061-7.
19. Hsu, T.; Speers, C.H.; Kennecke, H.F.; Cheung, W.Y. The utility of abbreviated patient-reported outcomes for predicting survival in early stage colorectal cancer. *Cancer* **2017**, *123*, 1839–1847, https://doi.org/10.1002/cncr.30511.
20. Kargo, A.S.; Jensen, P.T.; Lindemann, K.; Hjollund, N.H.I.; Lund, B.; Haee, M.; Möller, S.; Hansen, D.G.; Steffensen, K.D. The PROMova study comparing active and passive use of patient-reported outcome measures in ovarian cancer follow-up: effect on patient-perceived involvement, satisfaction with care, and usefulness. *Acta Oncol.* **2021**, *60*, 434–443, https://doi.org/10.1080/0284186x.2021.1891281.
21. Lagendijk, M.; Mittendorf, E.; King, T.A.; Gibbons, C.; Pusic, A.; Dominici, L.S. Incorporating Patient-Reported Outcome Measures into Breast Surgical Oncology: Advancing Toward Value-Based Care. *Oncol.* **2019**, *25*, 384–390, https://doi.org/10.1634/theoncologist.2019-0355.
22. Marino, D.; Baratelli, C.; Guida, G.; Turco, C.G.C.; Lacidogna, G.; Sperti, E.; Vignani, F.; De Luca, E.; Zichi, C.; Audisio, M.; et al. Impact of adoption of patient-reported outcomes in clinical practice on the accuracy of symptom reporting in medical records of cancer patients.. **2020**, *111*, 740–748.
23. McGee, S.F.; Zhang, T.; Jonker, H.; Laurie, S.A.; Goss, G.; Nicholas, G.; Albaimani, K.; Wheatley-Price, P. The Impact of Baseline Edmonton Symptom Assessment Scale Scores on Treatment and Survival in Patients With Advanced Non–small-cell Lung Cancer. *Clin. Lung Cancer* **2017**, *19*, e91–e99, https://doi.org/10.1016/j.cllc.2017.05.018.
24. Mooney, K.H.; Beck, S.L.; Friedman, R.H.; Farzanfar, R.; Wong, B. Automated monitoring of symptoms during ambulatory chemotherapy and oncology providers' use of the information: a randomized controlled clinical trial. *Support. Care Cancer* **2014**, *22*, 2343–2350, https://doi.org/10.1007/s00520-014-2216-1.
25. Montgomery, N.; The Cancer Care Ontario Patient Reported Outcome Advisory Committee; Howell, D.; Ismail, Z.; Bartlett, S.J.; Brundage, M.; Bryant-Lukosius, D.; Krzyzanowska, M.; Moody, L.; Snyder, C.; et al. Selecting, implementing and evaluating patient-reported outcome measures for routine clinical use in cancer: the Cancer Care Ontario approach. *J. Patient-Reported Outcomes* **2020**, *4*, 1–9, https://doi.org/10.1186/s41687-020-00270-1.
26. Nicolaije, K.A.H.; Ezendam, N.P.M.; Vos, M.C.; Pijnenborg, J.; Boll, D.; Boss, E.A.; Hermans, R.H.M.; Engelhart, K.C.M.; Haartsen, J.E.; Pijlman, B.M.; et al. Impact of an Automatically Generated Cancer Survivorship Care Plan on Patient-Reported Outcomes in Routine Clinical Practice: Longitudinal Outcomes of a Pragmatic, Cluster Randomized Trial. *J. Clin. Oncol.* **2015**, *33*, 3550–3559, https://doi.org/10.1200/jco.2014.60.3399.
27. Palmer, S.C.; Stricker, C.T.; DeMichele, A.M.; Schapira, M.; Glanz, K.; Griggs, J.J.; Jacobs, L.A. The use of a patient-reported outcome questionnaire to assess cancer survivorship concerns and psychosocial outcomes among recent survivors. *Support. Care Cancer* **2017**, *25*, 2405–2412, https://doi.org/10.1007/s00520-017-3646-3.
28. Richards, H.S.; Blazeby, J.; Portal, A.; Harding, R.; Reed, T.; Lander, T.; Chalmers, K.A.; Carter, R.; Singhal, R.; Absolom, K.; et al. A real-time electronic symptom monitoring system for patients after discharge following surgery: a pilot study in cancer-related surgery. *BMC Cancer* **2020**, *20*, 1–17, https://doi.org/10.1186/s12885-020-07027-5.
29. Rossi, L.A.; Melstrom, L.G.; Fong, Y.; Sun, V. Predicting post-discharge cancer surgery complications via telemonitoring of patient-reported outcomes and patient-generated health data. *J. Surg. Oncol.* **2021**, *123*, 1345–1352, https://doi.org/10.1002/jso.26413.
30. Smith, T.G.; Castro, K.M.; Troeschel, A.N.; Arora, N.K.; Lipscomb, J.; Jones, S.M.; Treiman, K.A.; Hobbs, C.; McCabe, R.M.; Clauser, S.B. The rationale for patient-reported outcomes surveillance in cancer and a reproducible method for achieving it. *Cancer* **2015**, *122*, 344–351, https://doi.org/10.1002/cncr.29767.

31. Smith, S.K.; Rowe, K.; Abernethy, A.P. Use of an electronic patient-reported outcome measurement system to improve distress management in oncology. *Palliat. Support. Care* **2013**, *12*, 69–73, https://doi.org/10.1017/s1478951513000345.
32. Stormoen, D.R.; Baeksted, C.; Taarnhøj, G.A.; Johansen, C.; Pappot, H. Patient reported outcomes interfering with daily activities in prostate cancer patients receiving antineoplastic treatment. *Acta Oncol.* **2021**, *60*, 419–425, https://doi.org/10.1080/0284186x.2021.1881818.
33. Stukenborg, G.J.; Blackhall, L.J.; Harrison, J.H.; Dillon, P.; Read, P.W. Longitudinal patterns of cancer patient reported outcomes in end of life care predict survival. *Support. Care Cancer* **2015**, *24*, 2217–2224, https://doi.org/10.1007/s00520-015-3024-y.
34. Suh, S.-Y.; Leblanc, T.W.; Shelby, R.A.; Samsa, G.P.; Abernethy, A.P. Longitudinal Patient-Reported Performance Status Assessment in the Cancer Clinic Is Feasible and Prognostic. *J. Oncol. Pr.* **2011**, *7*, 374–381, https://doi.org/10.1200/jop.2011.000434.
35. Sutradhar, R.; Rostami, M.; Barbera, L. Patient-Reported Symptoms Improve Performance of Risk Prediction Models for Emergency Department Visits Among Patients With Cancer: A Population-Wide Study in Ontario Using Administrative Data. *J. Pain Symptom Manag.* **2019**, *58*, 745–755, https://doi.org/10.1016/j.jpainsymman.2019.07.007.
36. Taarnhøj, G.A.; Johansen, C.; Lindberg, H.; Basch, E.; Dueck, A.; Pappot, H. Patient reported symptoms associated with quality of life during chemo- or immunotherapy for bladder cancer patients with advanced disease. *Cancer Med.* **2020**, *9*, 3078–3087, https://doi.org/10.1002/cam4.2958.
37. Takeuchi, E.E.; Keding, A.; Awad, N.; Hofmann, U.; Campbell, L.J.; Selby, P.J.; Brown, J.M.; Velikova, G. Impact of Patient-Reported Outcomes in Oncology: A Longitudinal Analysis of Patient-Physician Communication. *J. Clin. Oncol.* **2011**, *29*, 2910–2917, https://doi.org/10.1200/jco.2010.32.2453.
38. Van Egdom, L.S.E.; De Kock, M.A.; Apon, I.; Mureau, M.A.M.; Verhoef, C.; Hazelzet, J.A.; Koppert, L.B. Patient-Reported Outcome Measures may optimize shared decision-making for cancer risk management in BRCA mutation carriers. *Breast Cancer* **2019**, *27*, 426–434, https://doi.org/10.1007/s12282-019-01033-7.
39. Vistad, I. Electronic patient-reported outcomes to monitor symptoms after gynecological cancer treatment. *Acta Obstet. et Gynecol. Scand.* **2019**, *98*, 1365–1366, https://doi.org/10.1111/aogs.13734.
40. Wanat, M.; Walker, J.; Burke, K.; Sevdalis, N.; Richardson, A.; Mulick, A.; Frost, C.; Sharpe, M. Linked symptom monitoring and depression treatment programmes for specialist cancer services: protocol for a mixed-methods implementation study. *BMJ Open* **2017**, *7*, e016186, https://doi.org/10.1136/bmjopen-2017-016186.
41. Whitehead, L.; Emery, L.; Kirk, D.; Twigg, D.; Brown, D.; Dewar, J. Evaluation of a Remote Symptom Assessment and Management (SAM) System for People Receiving Adjuvant Chemotherapy for Breast or Colorectal Cancer: Mixed Methods Study. *JMIR Cancer* **2020**, *6*, e22825, https://doi.org/10.2196/22825.
42. Wright, A.A.; Raman, N.; Staples, P.; Schonholz, S.; Cronin, A.; Carlson, K.; Keating, N.L.; Onnela, J.-P. The HOPE Pilot Study: Harnessing Patient-Reported Outcomes and Biometric Data to Enhance Cancer Care. *JCO Clin. Cancer Informatics* **2018**, *2*, 1–12, https://doi.org/10.1200/cci.17.00149.
43. Yogananda, M.N.; Muthu, V.; Prasad, K.T.; Kohli, A.; Behera, D.; Singh, N. Utility of the revised Edmonton Symptom Assessment System (ESAS-r) and the Patient-Reported Functional Status (PRFS) in lung cancer patients. *Support. Care Cancer* **2017**, *26*, 767–775, https://doi.org/10.1007/s00520-017-3887-1.
44. Zebralla, V.; Müller, J.; Wald, T.; Boehm, A.; Wichmann, G.; Berger, T.; Birnbaum, K.; Heuermann, K.; Oeltze-Jafra, S.; Neumuth, T.; et al. Obtaining Patient-Reported Outcomes Electronically With "OncoFunction" in Head and Neck Cancer Patients During Aftercare. *Front. Oncol.* **2020**, *10*, https://doi.org/10.3389/fonc.2020.549915.
45. Zylla, D.M.; Gilmore, G.E.; Steele, G.L.; Eklund, J.P.; Wood, C.M.; Stover, A.M.; Shapiro, A.C. Collection of electronic patient-reported symptoms in patients with advanced cancer using Epic MyChart surveys. *Support. Care Cancer* **2019**, *28*, 3153–3163, https://doi.org/10.1007/s00520-019-05109-0.
